# A scalable method for discovering significant subnetworks

**DOI:** 10.1186/1752-0509-7-S4-S3

**Published:** 2013-10-23

**Authors:** Md Mahmudul Hasan, Yusuf Kavurucu, Tamer Kahveci

**Affiliations:** 1 Computer and Information Science and Engineering, University of Florida, FL 32611, USA; 2 Turkish Naval Academy, Istanbul, Turkey

## Abstract

**Background:**

Study of biological networks is an essential first step to understand the complex functions they govern in different organisms. The topology of interactions that define how biological networks operate is often determined through high-throughput experiments. Noisy nature of high-throughput experiments, however, can result in multiple alternative network topologies that explain this data equally well. One key step to resolve the differences is to identify the subnetworks which appear significantly more frequently in a biological network data set than expected.

**Method:**

We present a method named SiS (Significant Subnetworks) to find subnetworks with the largest probability to appear in a collection of biological networks. We define these subnetworks as the most probable subnetworks. SiS summarizes the interactions in the given collection of networks in a special template network. It uses the template network to guide the search for most probable subnetworks. It computes the lower and upper bound scores on how good the potential solutions are (i.e., the number of input networks that contain the subnetwork). As the search continues, it tightens the bound dynamically and prunes a massive number of unpromising solutions in that process.

**Results and conclusions:**

Experiments on comprehensive data sets depict that the most probable subnetworks found by SiS in a large collection of networks are also very frequent as well. In metabolic network data set, we found that subnetworks in eukaryote are more conserved than those of prokaryote. SiS also scales well to large data sets and subnetworks and runs orders of magnitude faster than an existing method, MULE. Depending on the size of the subnetwork in the same data set, the running time of SiS ranges from a few seconds to minutes; MULE, on the other hand, runs either for hours or does not even finish in days. In human transcription regulatory network data set, SiS finds a large backbone subnetwork that appears frequently regardless of diverse cell types.

## Introduction

Biological processes are administered by the complex interactions between different molecules. Such topology of interactions are regarded as *biological networks*. Systems biology aims to comprehend the biological processes that drive different functions in various organisms [1] through the study of biological networks. However, there are fundamental challenges in studying biological networks. Among those, two major challenges arise from (i) the noisy nature of the high-throughput experiments and (ii) the mathematical models used to infer the interactions from the experimental data. Measurement noise is inherent in high-throughput experiments. If we perform numerous high-throughput experiments even on the same cell line, each experiment can give rise to different measurements. This can potentially generate different sets of inferred interactions among these molecules. Several mathematical models are often used to reverse engineer the biological networks from the experimental data [2,3]. Given measurement data, mathematical model can infer different sets of interactions of the same quality resulting in alternative network topologies [4]. *One can identify a reliable backbone structure among these alternative network topologies by selecting the interactions that appear in significantly many of these networks*.

Graph is a mathematically robust representation that is often used to model biological networks. Given a graph *G *= (*V*, *E*), *V *is the set of nodes (with nodes corresponding to genes, proteins, enzymes etc.) and *E *is the set of directed edges (i.e., interactions between the nodes). Depending on the type of interaction, a graph can be directed or undirected. A number of interaction networks such as protein-protein interaction network are often modeled as undirected networks, whereas others such as transcription regulatory networks are often modeled as directed networks with the direction of edge denoting the regulatory relationship between the nodes. Undirected network is a special case of directed network model where each undirected edge is considered as a combination of two directed edges pointing opposite directions. Thus, the method described in this paper can be easily applied to undirected case as well. We defer formal definition of basic terminology to Section 3. Next, we briefly state the goal of the problem considered in this paper.

**Goal of this paper*** Consider two user supplied positive integers n and k. Also assume that we are provided with m alternative biological network topologies such that all the networks in this data set contain the same set of molecules. Let us denote the networks in this collection by graphs G_i _*= (*V*, *E_i_*), *for each value of i *= 1, 2, ... *m. Our goal is to find the n connected subgraphs of k edges that appear frequently in the given collection of networks*.

The problem tackled in this paper is similar to frequent subgraph discovery [5,6] and network motif identification [7,8] problems. These are computationally hard as these have deep connections with solving subgraph isomorphism (NP-Complete [9]) or graph isomorphism (GI-Complete [10]) problems, respectively. The complexity of the frequent subgraph discovery algorithm grows exponentially with increasing size of the subgraphs and input graphs. Several heuristic methods have been proposed to bound these solutions to practical execution time. These solutions have various limitations that reduce their use for many practical applications. Briefly, some of them find subgraphs only in a single network [11]. Among the methods that extend to multiple networks as input, some work only with smaller networks and subnetworks [12]. Some methods concentrate on the topology of the networks [13] and discount the content of the node altogether. We defer a discussion of the related literature to Section 2.

**Contributions** In this paper, we present *SiS *(**Si**gnificant **S**ubnetworks), an efficient method that finds frequent subnetworks in a given collection of biological networks. Assume that the user supplies two integers *n *and *k *and alternative biological network topologies. SiS starts by summarizing all the interactions in this collection in a hypothetical network called the *template network*. Template network is an edge-weighted network. The weight of each edge in this network equals to the minus logarithm of the fraction of the networks in the input data set which contain that edge. We assume that each network in the data set can be chosen with the same probability. SiS finds *n *connected subnetworks of *k *interactions with the highest probability to appear in a randomly drawn network (i.e., each of these subnetworks has smaller total edge weights than others in the template network). SiS exploits the use of template network in two ways: (i) it limits the search to the template network rather than every network in the data set and (ii) it prunes a massive number of unpromising solutions by using lower and upper bound values to the sum of the weights of all the edges in the tentative solution subnetworks. SiS also updates the upper bound values dynamically as it searches through the template network. Technical description of the method is presented in Section 3.

Using real metabolic network data sets and semi-synthetic network data sets, we evaluated the accuracy and the performance of our method. In our experiments, we observed that the frequent subnetworks of metabolic networks in eukaryote are more conserved than those of prokaryote. We also observed that subnetworks with large frequency in eukaryote also have large frequency in prokaryote. On the other hand, a substantial number of subnetworks with large frequency in prokaryote have very small frequency in eukaryote. We demonstrated experimentally that SiS is robust by simulating a broad spectrum of parameters determining the network topologies and result characteristics by testing it on the semi-synthetic data set. SiS was able to identify the true result accurately in our experiments. We also observed that SiS scales to large data sets easily. To find the maximal frequent subnetwork in global metabolic networks of eukaryote, SiS ran for less than five minutes; whereas, MULE [14], an existing method, finished in 7 hours and thirty minutes. SiS also discovers a large, core backbone network of interactions among human transcription regulatory networks of diverse cell types. Preliminary results of this work are published here [15].

We organize this paper as follows. We present the related works in literature in Section 2. We formally define the necessary terminology and describe the proposed solution SiS in Section 3 followed by the performance evaluation of SiS in Section 4. We conclude this paper in Section 5.

## Related work

One way to view the literature on the problem of discovering frequent subnetworks originates from two orthogonal perspectives. The first perspective focuses on the number of input networks (i.e., one or more); while the second one focuses on the labeling of the nodes and edges (i.e., labeled or unlabeled) of the input networks. From the first perspective, the problem has two variations:

(i) (Single input network) Here, the aim is to find subnetworks that appear the most number of times in a given large network [11,12].

(ii) (Multiple input networks) Here, the aim is to find subnetworks with each appearing at least once in a large number of networks in a given network data set. If a subnetwork exists in a network of the data set, its frequency increases by only one for each such network regardless of the number of copies in that network [5,6,14,16-19].

Many of the methods mentioned above disregard the possible labeling of the entities in the input network(s) and discover only the topology of interactions among the entities. This inevitably leads to the costly subgraph isomorphism problem. These methods differ from one another in the way that they consider nodes/edges of the input network(s). More importantly, they only focus on common topological features and ignore the contents (i.e., labels) of nodes and edges. *As a result, these common topological features may lead to subnetworks that have same topology but are not coherent in terms of the interactions or the interacting molecules*.

A number of algorithms discover frequent subnetworks by considering the labels of the interacting molecules. These algorithms bypass the subgraph isomorphism problem as the unique node labels make it trivial to find out if a given subnetwork is a part of a given large network. The challenge here is to decide which subnetworks should be explored as potential frequent subnetworks.

Among the above mentioned methods, MULE [14] discovers frequent subnetworks of enzyme interactions in a collection of metabolic networks. It models input networks as relational networks and represents each enzyme by a unique node label. CODENSE [18] seeks coherent dense subnetworks. It also models biological networks as relational networks. NEMO [20] reconstructs transcription regulatory modules in a systematic and efficient manner. Similar to CODENSE, MFC [21] searches for maximal frequent dense subnetworks in protein-protein interaction (PPI) networks.

In this paper, we consider multiple input networks where each network has uniquely labeled nodes. In the next section, we formally define the problem and our proposed solution.

## Proposed method

This section consists of the definition of common terminology (Section 3.1) followed by the description of the proposed method (Section 3.2).

### Preliminaries

**Figure 1:**
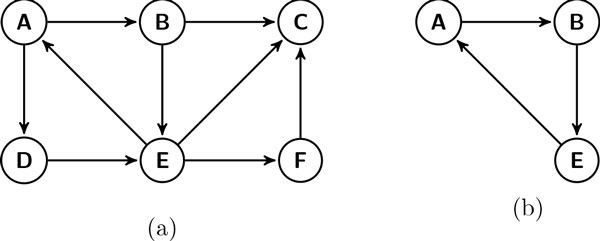
**A toy example**. (a) a graph *G *= (*V*, *E*) of six nodes and nine edges. Here, *V *denotes the set of nodes, {*A*, *B*, *C*, *D*, *E*, *F*} and *E *denotes the set of edges, {(*A*, *B*), (*A*, *D*), (*B*, *C*), (*B*, *E*), (*D*, *E*), (*E*, *A*), (*E*, *C*), (*E*, *F*), (*F*, *C*)} (b) a subgraph of three nodes.

In this section, we define a few terms and concepts that are used throughout this paper. A graph is a collection of nodes *V *connected by a set of edges *E*. We denote such a graph with *G *= (*V*, *E*). Given such a graph, *G *= (*V*, *E*), we call a graph *G' *= (*V'*, *E'*) to be a *subgraph *of *G *if *V' *is a subset of *V *and *E' *is a subset of *E *connecting the nodes in *V'*. We will use the terms graph and subgraph to denote network and subnetwork, respectively throughout this paper. In Figure 1 (a), we have a sample graph of six nodes and nine edges. Figure 1 (b) shows a subgraph of three nodes and three edges that appears in this sample graph.

An ordered sequence of nodes in a graph constitute a *path *in that graph, if all the consecutive node pairs are connected by an edge in this ordered sequence of nodes. For instance, In Figure 1 (a), nodes {A, D, E, F, C} make up a path as these nodes are incident to the edges {(A, D), (D, E), (E, F), (F, C)} in that order. We next define the concept of connectedness in a graph.

**Definition 1 **(Connected Node Pairs). *Given a graph G *= (*V*, *E*), *we say that nodes u and v (u*, *v *∈ *V*) *are connected if G contains a path that visits both nodes u and v*.

For instance, in Figure 1 (a), nodes *A *and *C *are connected as they both lie on path described above. We say that a *G *= (*V*, *E*) is a connected graph if all pairs of nodes (*u*, *v*), *u*, *v *∈ *V*, form connected node pairs.

This paper considers connected subgraphs only. This is mainly because in order the collections of genes to work together on a conserved function, such as regulating the transcription of certain sets of genes, they have to be connected in the underlying transcription regulatory network. We however emphasize that it is it is possible to formulate a disconnected subgraph as a set of connected subgraphs. That way, it is possible to extend the method developed in the rest of this paper to disconnected graphs as well.

In this paper, we denote a connected subgraph of *k *edges as a *size-k *subgraph. Thus, the number of nodes of a size-*k *subgraph is upper bounded by *k *+ 1, which is observed when the subgraph has a tree-like topology.

Consider the set of *m *graphs $D=\left\{G_{1},G_{2},.\;.\;.\;,G_{m}\right\}$. We first define the concept of *frequency *in  D.

**Definition 2 **(Frequency). *Assume that we are provided with a graph G' and a collection of graphs * D. *We define the frequency of G' over * D*(and denote it with *$f\left(G^{'},D\right)$*) as the number of graphs *$G_{i}\in \;D$*where G' is a subgraph of G_i_*.

In Definition 2, we describe the concept of frequency for the general case with any collection of graphs. In this paper, however, we assume that all the graphs in  D are built on the same collection of nodes, *V*, where each node in *V *has a unique label. Each graph $G_{i}\in D$ can thus be denoted with *G_i _*= (*V*, *E_i_*) such that ∀*i*, *E_i _*⊆ (*V × V*). Notice that, this assumption does not restrict the generality of Definition 2. To see that, consider a collection of *m *graphs  D in which different graphs may have different sets of nodes. In other words *G_i _*= (*V_i_*, *E_i_*) and ∃*G_i_*, $G_{j}\in \;D$ such that *i *≠ *j *and *V_i _*≠ *V_j_*. In that case, we can replace *G_i _*and *G_j _*with two new graphs $G_{i}^{'}$ and $G_{j}^{'}$ both containing the set of all nodes in *G_i _*and *G_j_*. In other words, $G_{i}^{'}=\left(V_{i}\cup V_{j},E_{i}\right)$ and $G_{j}^{'}=\left(V_{i}\cup V_{j},E_{j}\right)$. This operation, will make the two new graphs have identical node sets without altering their edge sets. By applying this operation repeatedly for all pairs of such graphs, we can create a data set with graphs, all containing the same set of nodes. Let us denote the resulting collection of graphs with $D^{'}$. Notice that all the new nodes in $G_{i}^{'}$ (i.e., nodes in the set *V_j _− V_i_*) are disconnected from the original nodes in *G' *(i.e., nodes in the set *V_i_*). Thus, those new nodes will never participate in any connected subgraph in $G_{i}^{'}$. This indicates that any subgraph with two or more nodes found in $D^{'}$ can also be found in  D. Thus, as we explain later in this paper expanding  D to $D^{'}$ has no effect in the outcome of our method.

Having defined the concept of frequency of a subgraph, we now formally define the concept of the most frequent subgraph in  D.

**Definition 3 **(Most Frequent Size-*k *Subgraph). *A size-k subgraph G in * D*is the most frequent size-k subgraph if *$f\left(G,D\right)\geq f\left(G^{'},D\right)$*for any size-k subgraph G' in * D.

It is possible to extend Definition 3 to multiple results. Following defines that extension.

**Definition 4 **(Top-*n *Most Frequent Size-*k *Subgraphs). *The top-n most frequent size-k subgraphs in * D*is the set of n subgraphs *$T_{n}$*with the largest frequency in * D. *Formally*, $∄G^{\prime }=\left(V^{\prime },E^{\prime }\right)\notin T_{n}$, *such that*

*(i) |E'| *= *k and*

*(ii) *$\exists G\in T_{n}$*for which *$f\left(G^{'},D\right)>f\left(G,D\right)$.

### SiS: Significant Subnetworks

We describe the proposed method SiS (Significant Subnetworks) in this section. We define the terminology specific to SiS and introduce a data structure needed to build it. We elaborate on different steps of SiS at the end of this section.

We start by considering the frequency of any edge *e *in  D. This is a special instance of Definition 2 with the subgraph *G' *limited to only one edge *e*. The frequency of an edge is bounded by the size of the input data set  D. Following definition eliminates this dependency.

**Definition 5 **(Relative Frequency of an Edge). *Relative frequency of an edge e in * D*(denoted by fr*(*e*,  D)*) is the frequency of that edge normalized by the number of networks in * D. *Formally, we compute the relative frequency as*

$$fr\left(e,D\right)=\frac{f\left(e,D\right)}{\left|D\right|}$$

Now, consider the following generative process. Assume that we randomly select a graph *G *= (*V*, *E*) from a given collection of graphs  D. We then randomly choose an edge *e *from *E*. Assume that we repeat this process *N *times. As *N *approaches to *∞ *the fraction of the generative iteration at which we observe *e *is equal to the relative frequency of *e *in  D. Similarly, consider the set of *k *edges *S *= {*e*_1_, *e*_2_, ..., *e_k_*} such that all edges in *S *belong to at least one graph in  D. Now, consider the following generative process to choose *k *edges from  D. At each iteration we first choose a graph *G *= (*V*, *E*) from  D. We then randomly choose an edge from *G*. Next, we remove *e *from all the graphs in  D and repeat this selection process until we choose a set of *k *edges. Let us denote the *N *sets of *k *edges obtained after applying this generative process *N *times with *S*_1_, *S*_2_, ..., *S_N_*. As *N *approaches to *∞*, the fraction of sets *S_i _*which are equal to *S *is equal to is $\underset{e_{i}\in \;S}{\prod }fr\left(e_{i},D\right)$. The more frequent the edges in *S *are, the higher the probability that they are selected by a randomized selection process.

To simplify the computation of the frequency, we use the following score function which is an equivalent representation to the frequency in our implementation:

$$score\left(G^{'},D\right)=-\text{log}\left(\underset{e\in E^{'}}{\prod }fr\left(e,D\right)\right)=-\underset{e\;\in \;E^{'}}{\sum }\text{log}\left(fr\left(e,D\right)\right)$$

Consider the top *n *most frequent size-*k *subgraphs ${Ḡ}^{1},{Ḡ}^{2},\;.\;.\;.\;,{Ḡ}^{n}$, in  D. Following from Definition 4, we know that it consists of frequent edges. In other words, all the edges *e *∈ *E' *have large fr(e,D) values. On the other hand, frequent edges in  D do not always jointly form frequent subgraph. With these observations in mind, consider the following generative process that randomly selects a size-*k *subgraph from  D. We first randomly select a graph G=(V,E)∈ D with replacement. Next, we randomly select a size-*k *subgraph of *G*, denoted with *G*^1^. At this point we have a randomly selected subgraph *G*^1^. Assume that, for a large number *N*, we repeat this generative process *N *times to select *N *subgraphs *G*^1^, *G*^2^, ..., *G^N ^*independently. We conjecture that for a randomly selected pair of indices *i*, *j *(*i *∈ [1 ... *n*] and *j *∈ [1 ... *N*]) the frequency of ${Ḡ}^{i}$ is at least as much as that of *G^j ^*with a high probability. Inversely, we say that a size-*k *subgraph *G *in  D is the most probable size-*k *subgraph if *score*(*G*) *≤ score*(*G'*) for any size-*k *subgraph *G' *in  D.

**Figure 2:**
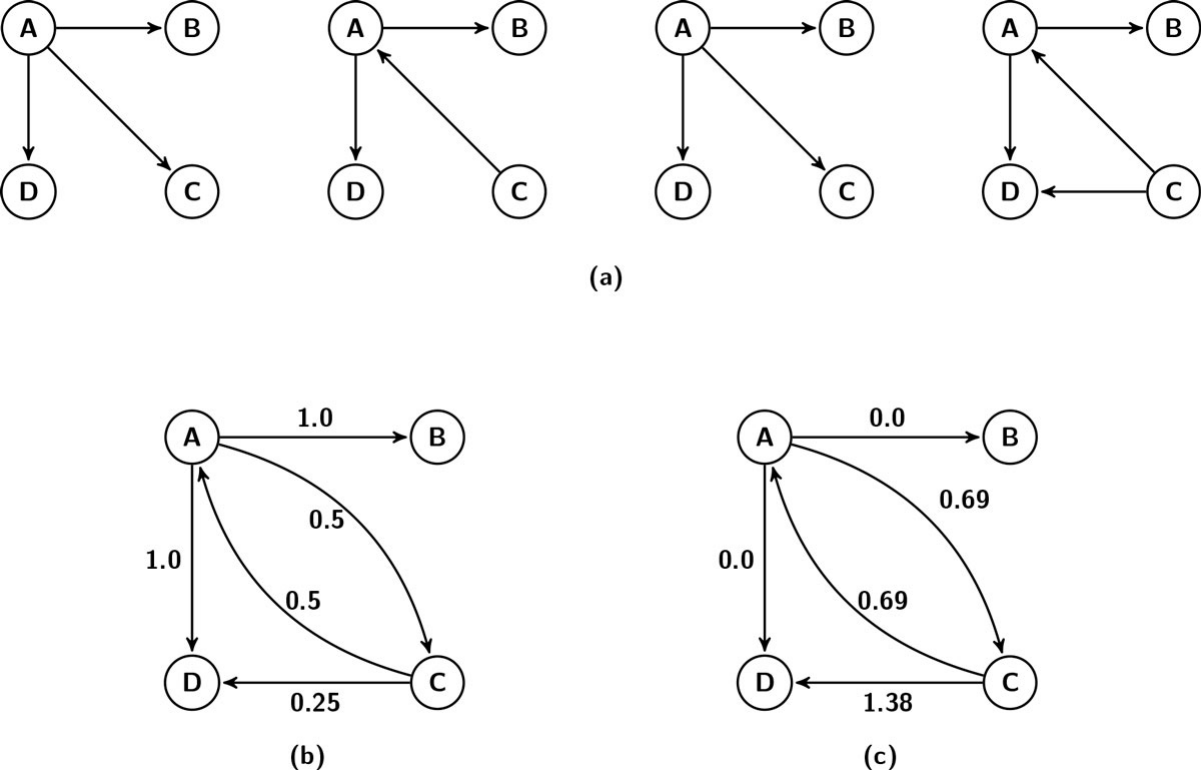
**A sample collection of input graphs and the resulting template graph**. (a) A collection  D of *m *= 4 graphs. Each graph has the same node set *V *= {*A*, *B*, *C*, *D*} but different edge set *E_i_*. (b) An edge-weighted graph summarized from  D. Weight for an edge *e *is fr(e,D). For example, the edge (*A*, *C*) appears in two graphs in  D, so, fr((A,C),D)=2/4=0.5. (c) The template graph for  D. Weight for an edge *e *is ϕ(e)=-log(fr(e,D)). For example, ϕ((A,C))=-log(fr((A,C),D))= - log(0.5)=0.69.

In this paper, we develop a method that finds the most probable subgraphs efficiently. Recall that each $G_{i}\in \;D$ has the same set of molecules; hence, can be represented as *G_i _*= (*V*, *E_i_*). We summarize all the graphs in  D using an edge weighted graph. We name this graph the *template graph*. Formally, the template graph of  D (denoted by *T *= (*V*, *E_T_*, *ϕ*())) is an edge weighted graph where *E_T _*is the set of all edges in  D, $\phi \left(\right):E_{T}\to \mathbb{R}$ and ϕ(e)=-log(fr(e,D)), ∀*e *∈ *E_T_*. Figure 2 shows an example with a sample collection of graphs and clarify the idea presented above.

**Figure 3:**
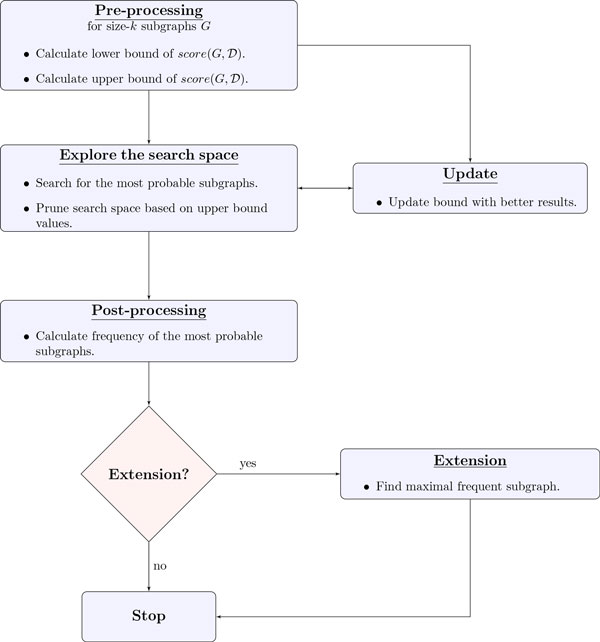
**A flowchart describing different steps of SiS**.

We design SiS to find most probable size-*k *subgraph in  D. SiS performs this task in several steps. Figure 3 shows a flowchart of SiS. By definition, the score value of the most probable subgraph is less than or equal to that of all the subgraphs in  D with the same number of edges. SiS calculates a lower and upper bound to this score by greedy technique. SiS then explores the search space and generates potential solution if its score is satisfied by the bound set in the previous step. As the search progresses, SiS narrows down the search space by pruning unpromising solutions. SiS also updates the upper bound value as better solution is found. The idea is to reduce the gap between the lower and upper bound and use this margin as a guide to find the optimal result. SiS continues to prune most of the search space using the heuristic mentioned above until the whole search space is explored. Next, we elaborate on each step of SiS.

**Pre-processing **In this step, we calculate lower bound and upper bound value of *score *$\left(G^{'},D\right)$ for most probable size-*k' *subgraph *G' *for each value of *k' *= 1, 2, ..., *k*. We explain these processes below.

**Lower bound calculation **A greedy technique is employed to calculate a lower bound to the value of *score*(*G'*, *D*) for any potential most probable size-*k' *subgraph *G' *for each value of *k' *= 1, 2, ..., *k*. It is impractical to generate all size-*k' *subgraphs and compute the minimum of their score values. Instead, the following scheme quickly obtains the desired score value: let us sort the edges in the template graph (i.e., *e *∈ *E_T_*) in non-decreasing order based on *ϕ*(*e*) values. Assume that the first *k' *edges in this sorted list form a connected subgraph *G″ *= (*V″*, *E″*). This relaxation allows a simple way to compute the lower bound values as *score *$\left(G^{\prime \prime },D\right)=\sum_{e\in E^{\prime \prime }}\phi \left(e\right)$ is guaranteed to be at least as small as *score *$\left(G^{'},D\right)$ of any size-*k' *subgraph in *T*. The following lemma formally presents the correctness of this idea.

**Lemma 1 **(LOWER BOUND). *The score of a size-k subgraph computed as in the above pre-processing step is a lower bound to the score of an optimal size-k subgraph*.

*Proof*. Let *G *be the size-*k *subgraph generated by the pre-processing step and G* be an optimal size-*k *subgraph. Let the *k *edges of *G *and G* in non-decreasing order of *ϕ*() values be represented by {*e*_1_, *e*_2_, ..., *e_k_*} and $\left\{e_{1}^{G*},e_{2}^{G*},\;.\;.\;.\;,e_{k}^{G*}\right\}$, respectively. Assume that the *score*(*G*) is not a lower bound to the *score *of an optimal size-*k *subgraph (i.e., *score*(*G*) *> score*(G*)). It implies that there is at least one edge in G* with lower *ϕ*() value with respect to the corresponding edge in *G*. Let *e_i _*be the first of such edges so that $\phi \left(e_{i}\right)>\phi \left(e_{i}^{G*}\right)$. However, there can be no such *e_i _*as the pre-processing step selects *k *edges with the smallest *ϕ*() values in the template graph, *T*. Therefore, the lower bound obtained in the pre-processing step is indeed a lower bound to the *score *of optimal subgraph. □

**Upper bound calculation **A greedy technique is employed to calculate the upper bound to *score *$\left(G^{'},D\right)$ for any potential most probable size-*k' *subgraph *G' *for each value of *k' *= 1, 2, ..., *k*. Our rationale is to generate upper bound values close to the optimal ones. We generate a size-*k' *subgraph by augmenting an edge to a size-(*k' − *1) subgraph. Assume that we initialize *G' *to an arbitrary edge *e *∈ *E_T _*to generate a size-1 subgraph. We get a size-2 subgraph by augmenting an edge *e' *to it such that *e' *is incident to a node in *G' *and *ϕ*(*e'*) is minimum among all such edges. In this manner, we can generate a size-*k' *subgraph starting from a size-1 subgraph. If we fix the starting edge *e*, we can easily determine the edges that will be selected subsequently. Therefore, the score of any size-*k' *subgraph completely depends on the choice of the initial edge, *e*. Our goal is to generate upper bound value that is not too far from the optimal one. We iterate over every edge *e *∈ *E_T_*, generate desired size-*k' *subgraph and select the minimum score of such subgraphs as the upper bound value. The following lemma delineates the correctness of the obtained upper bound to the optimal score in the pre-processing step.

**Lemma 2 **(UPPER BOUND). *The score of a size-k subgraph computed as in the above pre-processing step is an upper bound to the score of an optimal size-k subgraph*.

*Proof*. Let *G *be the size-*k *subgraph generated by the pre-processing step and G* be an optimal size-*k *subgraph. We prove by contradiction that *score*(*G*) *≥ score*(G*), i.e., *score*(*G*) is an upper bound to *score*(G*). By definition, *score*(G*) is the smallest among the scores of all possible size-*k *subgraphs. Therefore, *score*(*G*) is either equal to optimal value (i.e., *score*(*G*) = *score*(G*)) or greater than the optimal value (i.e., *score*(*G*) *> score*(G*)). Therefore, the upper bound obtained in the pre-processing step is indeed an upper bound to the *score *of optimal subgraph. □

We will see how we use these bounds in later stages of the algorithm to filter large part of the search space.

**Explore the search space **In the pre-processing step, we calculate the lower and upper bound values for most probable size-*k' *subgraph for each value of *k' *= 1, 2, ..., *k*. We store these score values of lower and upper bound in *L*[1 ... *k*] and *U*[1 ... *k*] arrays, respectively. Here, *L*[*i*] and *U*[*i*] entries store the lower bound and upper bound of score values of the size-*i *subgraph, respectively. Next, We describe how SiS incorporates the bounds calculated in the pre-processing step to accelerate the search process.

SiS enumerates the subgraphs of the template graph incrementally, i.e., by extending smaller subgraphs to larger subgraphs. Suppose that *G' *= (*V'*, *E'*) was initialized as a size-1 subgraph where *E' *= {*e_i_*} (*e_i _*is an arbitrary edge ∈ *E_T_*) and *V' *consists of the nodes incident to *e_i_*. Assume that SiS extends this to a size-*k' *subgraph *G' *now. One can further augment *G' *to a size-(*k' *+ 1) subgraph *G″ *by adding a new edge *e_j _*to *E'*. This can be done if the following constraints are satisfied:

(i) *e_j _*is incident to a node *v *∈ *V'*.

(ii) The index of the starting edge is smaller than that of the new edge (i.e., *i < j*).

(iii) *score *$\left(G^{\prime \prime },D\right)+L\left[k-k^{'}-1\right]\leq U\left[k\right]$.

Now, let us elaborate on the roles of these constraints. The first one above guarantees that the generated subgraph remains connected as we add one of the incident edges of the smaller subgraph. The second one is not necessary for correctness of the algorithm; it rather keeps the execution time of SiS practical. It ensures that SiS never generates and evaluates the same subgraph twice. The last constraint is used to filter a massive set of unpromising subgraphs from the search space. If this fails, SiS discards the current solution as it will not lead to the optimal result. In other words, unsuccessful evaluation of this constraint denotes that SiS already generated a better solution and its score is stored in the corresponding entry of the upper bound array *U *. Note that the subgraph generation strategy described above depends on the initial choice of *e_i_*. SiS repeats this search process by iterating over every *e *∈ *E_T _*one by one.

Recall that by definition, the most probable size-*k' *subgraph has the smallest score value among all the size-*k' *subgraphs. So, the score of any size-*k' *subgraph is actually an upper bound to the optimal score value. Thus, during the search process, if SiS generates a size-*k' *subgraph *G' *whose *score*(*G'*) *< U*[*k'*], we set *U*[*k'*] to *score*(*G'*). Thus, *U*[*k'*] always stores the score of the best size-*k' *subgraph generated so far. Dynamically updating the upper bound values while exploring the search space contributes instantly to the performance of the search process. It leads to further pruning of more unpromising solutions. As showed in later experiments, this empowers SiS scale to very large graphs and subgraphs.

The method described above searches for the most probable size-*k' *subgraph for each value of *k' *= 1, 2, ..., *k*. It is worth mentioning that extending it to find the *n *most probable subgraphs is trivial. We store the upper and lower bounds to the top-*n *size-*k' *subgraphs for each value of *k' *= 1, 2, ..., *k*. We should also extend this idea accordingly for constraint (iii) mentioned above. In Section 4.2, we report the performance of SiS for different values of *n *and *k*.

**Post-processing and extensions **SiS computes the frequency of the *n *most probable subgraphs found in the previous step. Recall that the input graphs are labeled where label of a node can denote the name of the gene, enzyme or protein, for instance. It is computationally inexpensive to check the existence of a subgraph when both graph and subgraph are labeled. To compute the frequency of a subgraph, we need to iterate over all the input graphs. So, we repeat this process for each of the *n *most probable subgraphs.

We can easily augment SiS to find maximal frequent subgraphs in  D. A maximal frequent subgraph is a frequent subgraph that is not a subgraph of any frequent subgraph. We employ SiS to find maximal frequent subgraph in the following manner. First, SiS generates *n *most probable size-*k *subgraphs. It uses moderately large values of *n *and *k*. As most probable subgraphs have large frequency values (elaborated in Section 4), they serve as good seeds for the greedy technique. For each of the most probable subgraph, we augment an edge to it at a time if the new subgraph is both connected and frequent. This is a simple greedy extension technique to generate larger subgraphs with frequency values greater than a given threshold. In Sections 4.4 and 4.5, we describe the performance of SiS as a tool to find maximal frequent subgraph.

## Experiments

This section evaluates the performance of SiS experimentally. We used a comprehensive set of both real and semi-synthetic data sets. Using real data set, we employed SiS to find out how frequent the metabolic networks in eukaryote and prokaryote are. We generated the maximal frequent subgraph in human transcription regulatory networks. Using semi-synthetic data set, we evaluated the robustness of SiS simulating many different experimental conditions. We also compared SiS with a maximal frequent subgraph finding algorithm, MULE using real data set. We describe the data sets below.

**Real data set **We used two types of biological network data sets, namely (i) metabolic networks and (ii) human transcription factor regulatory networks. Let us elaborate on these data sets first. We downloaded all the metabolic networks from KEGG database [22]. We created two separate data sets for eukaryote and prokaryote in all cases. Let *u *and *v *be two enzymes in a metabolic network and *u *catalyzes a reaction *r*_1 _and *v *catalyzes a reaction *r*_2_. Assume that *r*_2 _consumes a product produced by *r*_1_. We denote this relationship by a directed edge (*u*, *v*). We represent an enzyme by a single node in this model even if it catalyzes multiple reactions in a pathway. Each organism may employ a marginally different set of enzymes to perform the same function (e.g., carbohydrate, lipid, energy metabolism etc.). To ensure that each organism in a data set is built on the same set of enzymes, we set it to the union of all enzymes in the data set. We created the *global network *data set from global metabolic pathways (i.e., Eukaryote-G and prokaryote-G). As an example of nucleotide metabolism, we created a small data set from pyrimidine metabolism (i.e., Eukaryote-P and prokaryote-P). Similarly, for amino acid metabolism, we created a small data set from alanine, aspartate and glutamate metabolism (i.e., Eukaryote-AAG and prokaryote-AAG). To measure the potency of SiS, we also used human transcription regulatory networks data among 41 diverse cell and tissue types (for example, pulmonary artery fibroblast (HPAF), astrocyte (NHA), fetal heart (fHeart) etc.) [23]. Table 1 presents details on the real data sets.

**Table 1:** An overview of the real data sets used in the experiments.

Data set	Number of Networks	Entire Data set		Template Graph	
		
		Nodes	Edges	Nodes	Edges
Eukaryote-G	145	45,315	78,499	1,413	1,541
Prokaryote-G	1,486	393,681	616,546	1,413	1,676
Eukaryote-AAG	145	2,048	2,951	43	54
Prokaryote-AAG	1,442	20,692	27,334	43	79
Eukaryote-P	145	2,942	5,757	63	103
Prokaryote-P	1,486	31,130	60,802	63	114
HTRN	41	21,377	574,122	538	47,945

Suffixes *G, AAG *and *P *stand for *Global, Alanine, Aspartate and Glutamate *and *Pyrimidine*, respectively. HTRN denotes Human Transcription Regulatory Networks [15].

**Semi-synthetic data set **We downloaded five different signaling networks (*VEGF*, *Apoptosis*, *WNT*, *ERB *and *MAPK*) of human (*Homo sapiens*) from KEGG database. By augmenting these networks, we generated one composite network of 236 nodes and 348 interactions. We then generated a collection  D of *m *= 100 graphs from this single composite network. We used degree-preserving mutation [24] with a given mutation rate (*µ*) to generate each of these input graphs $G_{i}\in \;D$. In order to verify how well our algorithm performs under several conditions, we purposefully implanted subgraphs of a given size (*k*) and frequency (*f *)% in  D as follows. We first randomly selected a size-*k *subgraph from the composite graph. We then ensured that this subgraph remained unaltered in at least *f *% of the input graphs $G_{i}\in \;D$ by marking the edges of this subgraph in *f *% of the *G_i_s*. In this way, we simulated different experimental conditions by creating data sets with varying size (*k*) of the implanted subgraph, mutation rate (*µ*) and frequency (*f *).

**Implementation and environment **We implemented SiS in *C*++ and tests are performed on a computer equipped with Intel Core i7 2.67 GHz CPU, 4 GBs of main memory running Windows 7 operating system.

### Subgraphs in metabolic network data set

In these experiments, we investigate how conserved the metabolic networks are and how the frequent subgraphs in eukaryote and prokaryote relate to each other. In experiments described in this section, we first employed SiS to find 50 most probable size-*k *subgraphs for different values of *k*. Then we calculated the actual frequency of these subgraphs.

**Figure 4:**
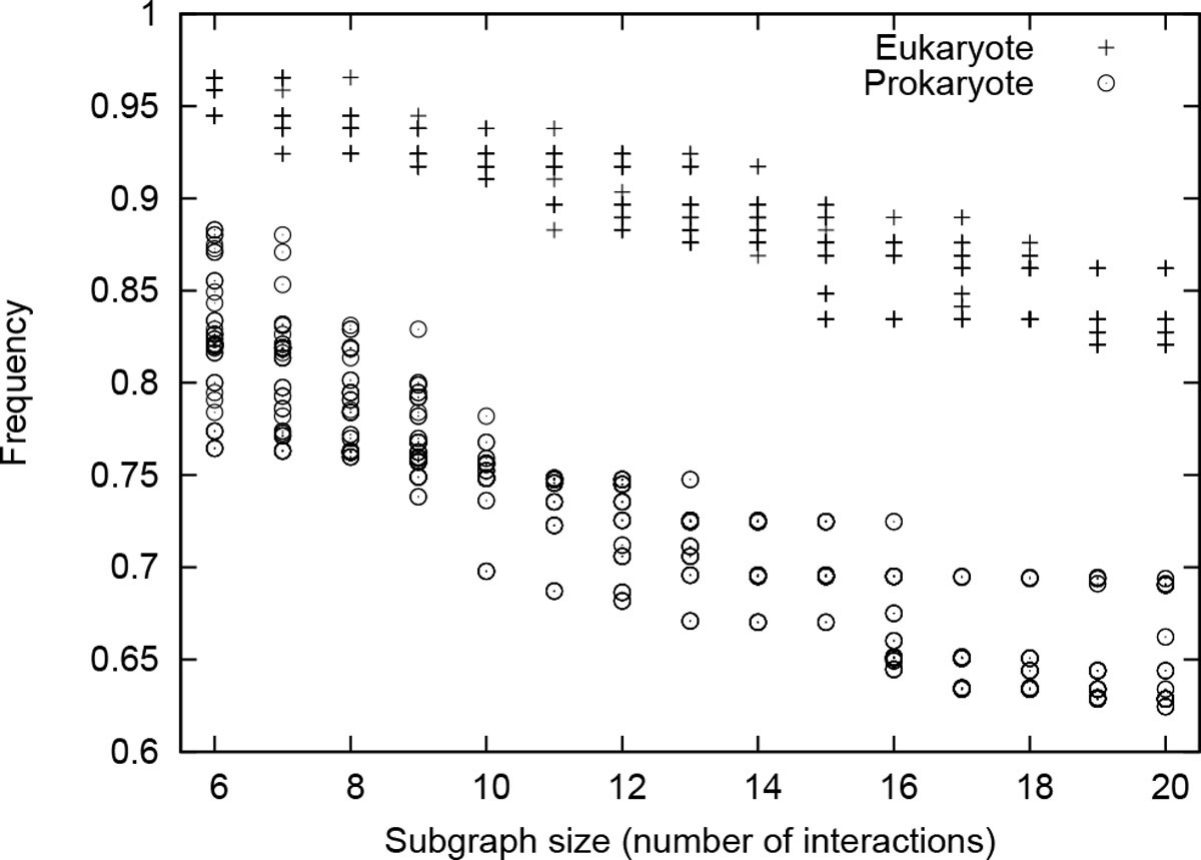
**Normalized frequency of the top 50 most probable size-*k *subgraphs for each value of *k *= 6, 7, ..., 20 in Eukaryote-G and Prokaryote-G data sets **[15].

**How conserved the enzyme subnetworks in eukaryote and prokaryote are **In this experiment, we employed SiS to find most probable size-*k *subgraphs for each value of *k *= 6, 7, ... 20 in both Eukaryote-G and Prokaryote-G data sets. This generated 750 (i.e., 50 *× *(20 - 5)) subgraphs in total for each of these data sets. Figure 4 plots the frequency of the generated most probable subgraphs for both these data sets. High frequency value of the subgraphs exhibits the presence of large and highly conserved subgraphs in eukaryote and prokaryote. These highly conserved subgraphs denote that both eukaryote and prokaryote generally use the same set of interactions to perform their functions. Figure 4 also shows that the frequency of the most probable subgraphs in eukaryote are higher than those of prokaryote even for small subgraphs. This is well established as eukaryote displays functional conservation at both protein as well as network level [25]. We also observe a gradual drop in frequency for subgraphs in both clades as the size of the subgraph increases. This implies that there are additional interactions (apart from those in the subgraph) that appear in many organisms. Otherwise, increase in subgraph size will result in exponential decrease in the frequency of that subgraph.

**Figure 5:**
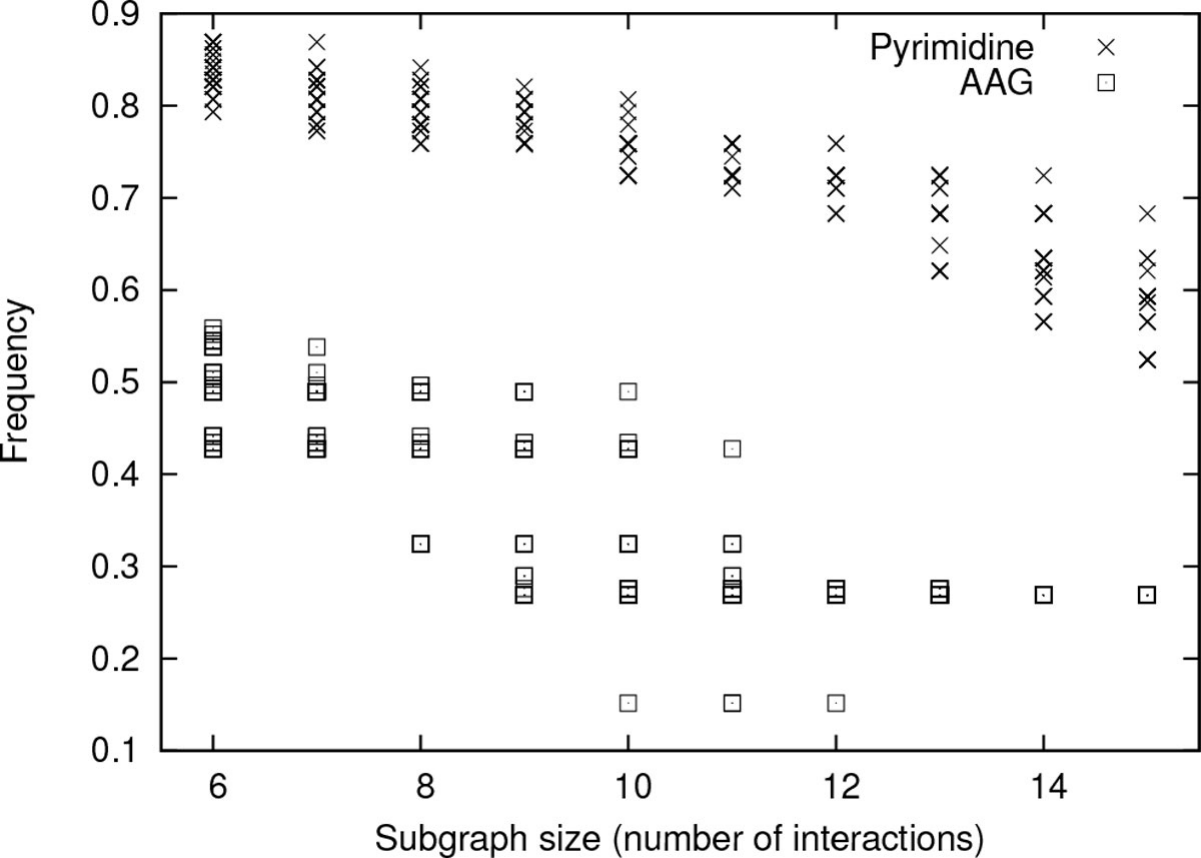
**Normalized frequency of the top 50 most probable size-*k *subgraphs for each value of *k *= 6, 7, ..., 15 in Eukaryote-P and Eukaryote-AAG data sets **[15].

**Figure 6:**
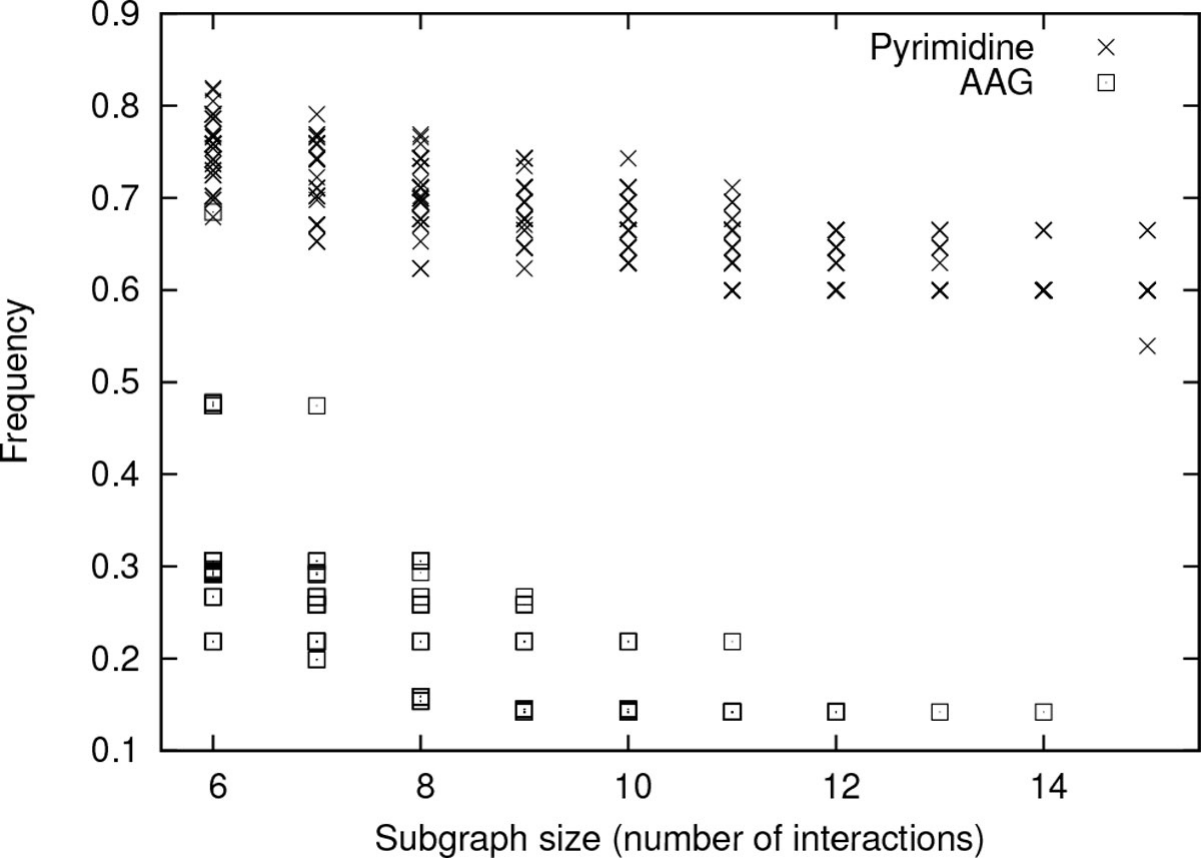
**Normalized frequency of the top 50 most probable size-*k *subgraphs for each value of *k *= 6, 7, ..., 15 in Prokaryote-P and Prokaryote-AAG data sets**.

**Conserved enzyme subnetworks in pyrimidine and alanine, aspartate, glutamate networks **In this experiment, we used smaller metabolic network data sets (i.e., Eukaryote-AAG, Prokaryote-AAG, Eukaryote-P and Prokaryote-P). We employed SiS to find most probable size-*k *subgraphs for each value of *k *= 6, 7, ... 15. Figures 5 and 6 plot the frequency of these subgraphs in eukaryote (using Eukaryote-AAG and Eukaryote-P data sets) and prokaryote (using Prokaryote-AAG and Prokaryote-P data sets), respectively. In both eukaryote and prokaryote, pyrimidine network is found to be significantly more conserved than alanine, aspartate and glutamate network. The most probable subgraphs found by SiS in pyrimidine network are present over 50% of the organisms in both eukaryote and prokaryote even for size-15 subgraphs. We also observe that the frequency starts to drop as the size of the subgraph increases. This drop is gradual in pyrimidine network. However, in alanine, aspartate and glutamate network, the frquency of the most probable subgraphs of size-*k *are small for large *k *values (e.g., in Prokaryote-AAG data set, most probable size-*k *subgraphs are very infrequent for *k ≥ *11).

**Figure 7:**
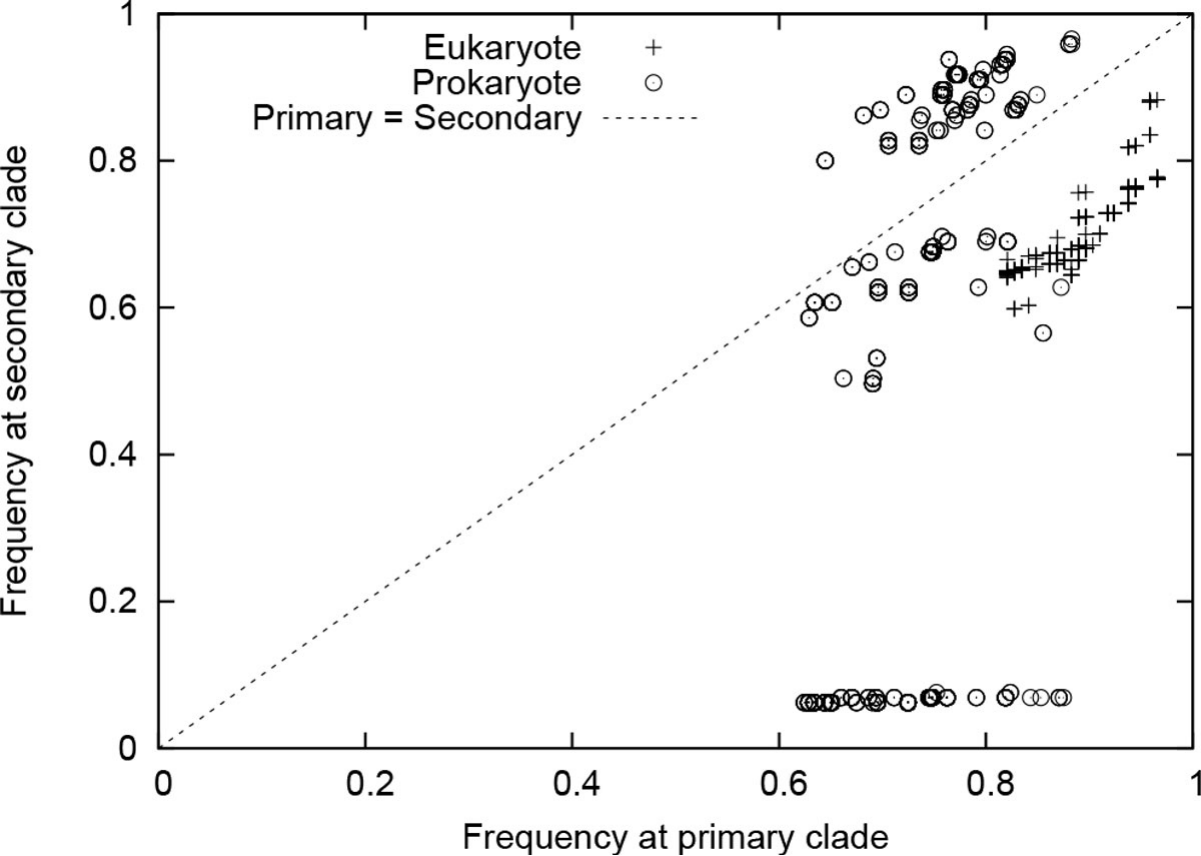
**Normalized frequency of the top 50 most probable size-*k *subgraphs for each value of *k *= 6, 7, ..., 20 in primary (eukaryote/ prokaryote) and corresponding secondary clade (prokaryote/ eukaryote) using Eukaryote-G and Prokaryote-G data sets **[15].

**Figure 8:**
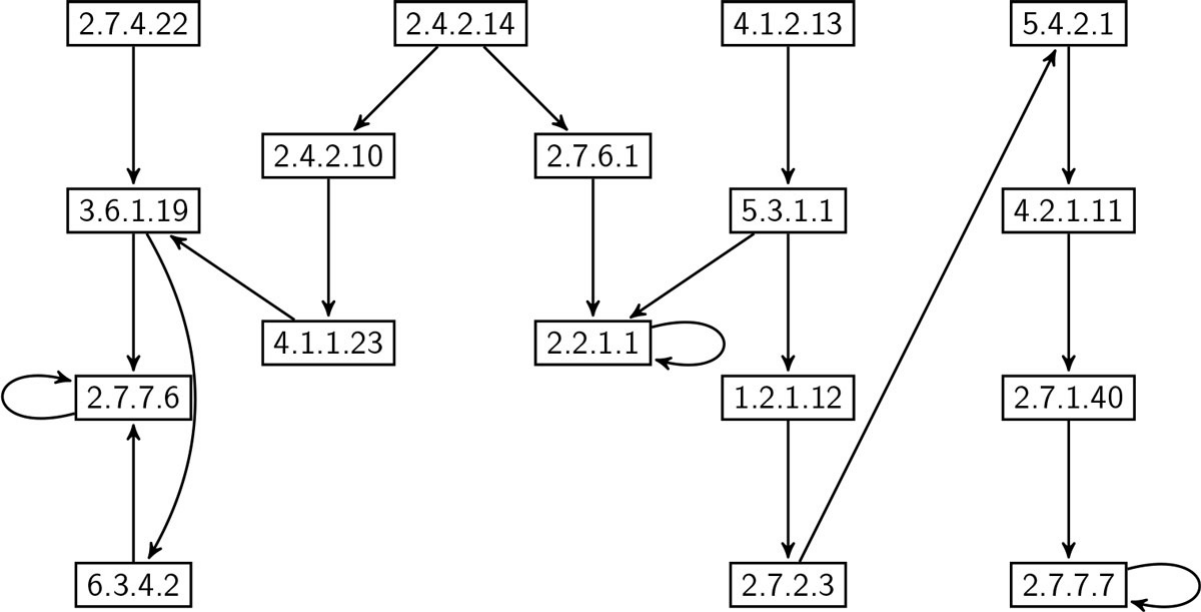
**A size-20 subgraph which is highly frequent (69%) in prokaryote, but infrequent (7%) in eukaryote**. Node label refers to Enzyme Commission (EC) number [15].

**Figure 9:**
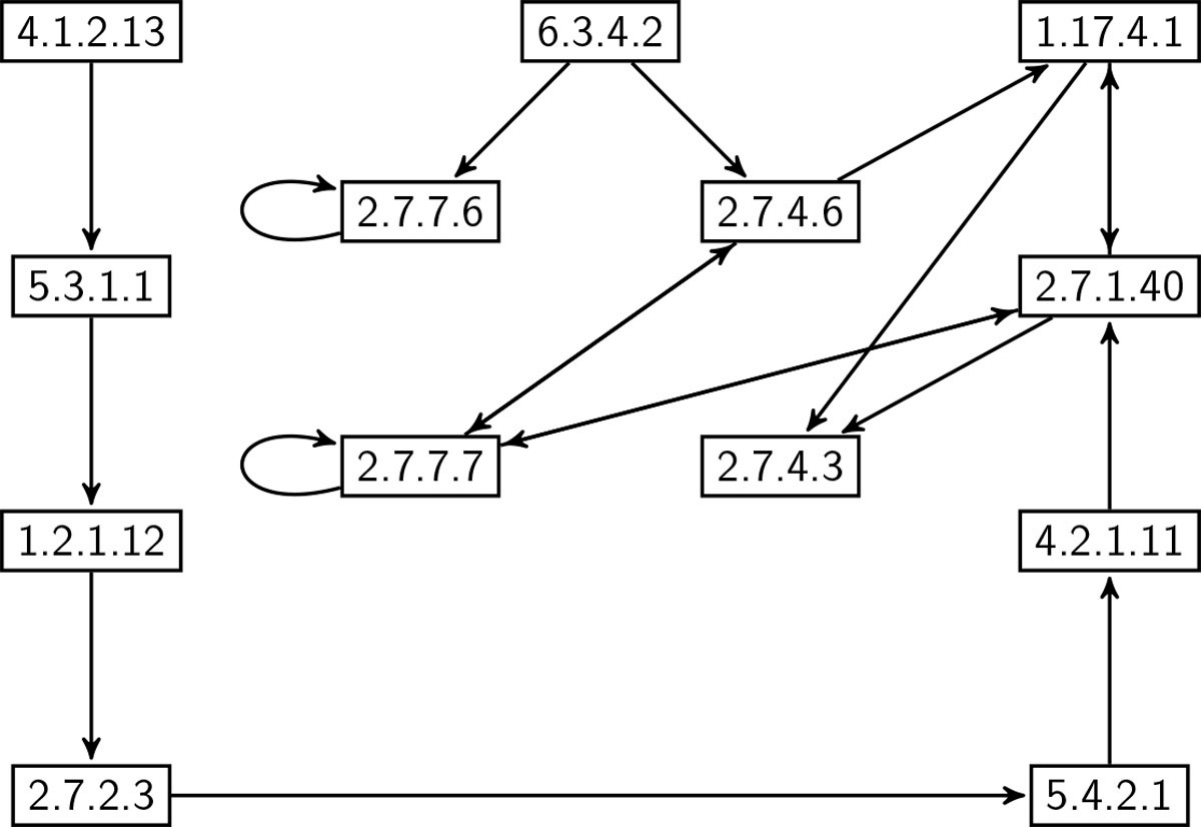
**A size-20 subgraph which is frequent in both eukaryote (86%) and prokaryote (66%)**. Node label refers to Enzyme Commission (EC) number [15].

**Correlation of frequent enzyme subnetworks in eukaryote and prokaryote **In this experiment, we evaluate the correlation between frequent subgraphs in eukaryote and prokaryote using global network data set. We employed SiS to find most probable size-*k *subgraphs for each value of *k *= 6, 7, ..., 20 in both eukaryote and prokaryote. For each of these subgraphs, we calculated their frequency in both data sets. We denote the data set where SiS identified these subgraphs as *primary clade*; the other one is denoted by *secondary clade*. Thus, we computed two frequency values for each resulting subgraph. It shows how frequent one subgraph found in primary clade is in secondary clade. Figure 7 plots the frequency values of these subgraphs. It shows that the most probable subgraphs found in eukaryote are also frequent in prokaryote. However, plots in the lower right of the figure demonstrates that there are some frequent subgraphs in prokaryote that are very infrequent in eukaryote. This observation is not unexpected as almost all metabolic pathways in eukaryote are also present in prokaryote [26]. However, some prokaryote, especially bacteria and archaea living in extreme environments, are known to have indigenous pathways. For instance, prokaryote manage anaerobic respiration [27] and produce methane through metanogenesis [28] using pathways that are either absent or deviate from eukaryote. Figure 8 shows a subgraph that is frequent in prokaryote but very infrequent in eukaryote. Figure 9, on the other hand, shows a subgraph that is frequent in both eukaryote and prokaryote. These two size-20 subgraphs contain 17 and 13 enzymes, respectively. Interestingly, these two subgraphs have 10 enzymes and 10 interactions that are common. The remaining enzymes and interactions play a vital role in the striking difference in frequency. For instance, prokaryote use UMP kinase (EC number: 2.7.4.22) for synthesis of pyrimidines, whereas eukaryote use UMP/CMP kinase (EC number: 2.7.4.14) for the same purpose.

### Subgraphs in semi-synthetic data sets

In this section, we test the robustness of SiS under different experimental conditions. In this respect, we generated different semi-synthetic data sets by varying a number of parameters to simulate different conditions. We implanted a subgraph of certain size (*k*) and frequency (*f *)%; then generated a number of data sets by mutation (*µ*) of the interactions. We employed our method to discover the implanted subgraph from this data set. We experimented with different size (*k*) of the implanted frequent subgraph, mutation rate (*µ*) and frequency (*f *)%. We tested SiS on these data sets to verify that the discovered subgraphs had frequency values at least as much as of the implanted subgraph. We repeated each experiment 10 times for SiS and report the average results.

**Impact of mutation rate **In the first set of experiments, we examined the influence of mutation rate (*µ*) on *D *for SiS. We implanted a size-15 subgraph with 70% frequency and changed the mutation rate, *µ *to 5%, 10%, 20%, 30% and 40% to generate *D*. Recall that large values of *µ *implies more deviation in the topologies of the input graphs. Table 2 summarizes the average frequency of the most probable size-15 subgraph discovered by SiS in *D *with respect to above-mentioned mutation rates. In these experiments, we found that in data sets with *µ ≥ *20%, the only subgraph with more than 70% frequency was the subgraph that we implanted. It shows that SiS is capable of finding frequent subgraphs even in data sets with high mutation rate. This supports our earlier conjecture that most probable subgraphs and most frequent subgraphs often are the same or comparable in terms of their frequency.

**Table 2:** Frequency of the most probable size-15 subgraph found by SiS for various mutation rates.

Mutation rate (%)	5	10	20	30	40
Frequency reported by SiS (%)	84.9	73.5	71.9	71.2	71.1

The frequency of the implanted subgraph was 70% for all mutation rates.

**Impact of frequency value **Next, we experimented by varying the frequency value (*f *) of the implanted subgraph. For these experiments, we implanted a size-15 subgraph with frequency value, *f *set to 50%, 60%, 70%, 80%, 90% and 95%. In these data sets, we fixed the mutation rate, *µ *at 10%. Table 3 presents the average frequency value of the most probable subgraph discovered by SiS in  D and that of implanted subgraph. We observe that the frequency of the most probable subgraph we found was at least as good as that of the implanted one on the average for all parameter settings. The larger observed frequency values imply that there were additional subgraphs aside from the implanted ones that contained the same subgraph and SiS was able to locate them. In our experiments, SiS failed to find the implanted subgraph in only a few of the 60 data sets (i.e., 10 data sets per implanted frequency value). This happened only when frequency values of the implanted subgraphs were 70% or more. In these few data sets, the gap between the frequency reported by SiS and that of the implanted one was about 2% or less. In these cases, the most probable subgraphs differ from the most frequent ones, but their frequency values are still very much comparable.

**Table 3:** Frequency of the most probable size-15 subgraph found by SiS for various frequency values.

Frequency of the planted subgraph (%)	50	60	70	80	90	95
Frequency reported by SiS (%)	58.0	66.3	73.1	82.6	92.5	96.5

Mutation rate was fixed at 10% for all the frequency values.

**Impact of the size of implanted subgraph **We also experimented with implanted subgraphs of varying sizes (*k*). In these experiments, we implanted a subgraph of size 5, 10, 15, and 20, respectively with frequency, *f *= 70% and mutation rate, *µ *= 10%. The result is shown in Table 4 for the most frequent subgraph. We observe that the frequency of the most probable subgraph found was at least as good as that of the implanted one on the average for all parameter settings. The larger observed frequency values for smaller implanted subgraphs (*k ≤ *10) imply that there were additional subgraphs aside from the implanted ones that contained the same subgraph and SiS was able to locate them. For relatively larger subgraphs (*k ≥ *15), frequency of the most probable subgraph was just above the frequency of the implanted ones. It implies that when the size of the implanted subgraph grows, with the current parameter settings, the subgraphs discoverd are the implanted ones. This experiment demonstrates that SiS can discover frequent subgrahs succesfully even for very large sized subgraphs.

**Table 4:** Frequency of the most probable size-*k *subgraph found by SiS for different sizes of the implanted subgraph.

Size of implanted subgraph (*k*)	5	10	15	20
Frequency reported by SiS (%)	88.1	78.6	71.7	70.4

The frequency of the implanted subgraph was 70% for all subgraph sizes.

*In summary, our experiments in this section demonstrate that SiS can identify most frequent subgraph for a broad set of parameter values*.

### Running time performance

A frequent subgraph discovery method is practical only if it scales to the size of the network and subnetwork. In this section, we evaluate the running time of SiS and compare it with exhaustive search. For a fair comparison with SiS, we ran our exhaustive search implementation on the template graph (rather than on every input graph in  D and finding the corresponding frequency over  D). We compare the running time of SiS and exhaustive search for various size of subgraph (*k*). To generate the semi-synthetic data sets for this set of experiments, we implanted a subgraph of size-*k *with frequency, *f *= 70% and fixed the mutation rate, *µ *= 10%. We used a frequency threshold of 5% (i.e., in  D of *m *= 100 networks, we searched for subgraph that existed at least in 5 of them). The running times of SiS and exhaustive search are given in Table 5. It shows that SiS finds frequent subgraphs containing up to 20 interactions very fast.

**Table 5:** Running time of SiS and exhaustive search for semi-synthetic data set.

Subgraph size (*k*)	6	7	8	9	15	20
SiS	0.02	0.02	0.02	0.2	1.78	33.24

Exhaustive	43.33	421.20	7535.10	-	-	-

The time measurements are in seconds. "-" means that the experiment did not finish to completion in over two days.

*The results clearly demonstrate that our method scales to large subgraph sizes when exhaustive search quickly becomes infeasible*. We elaborate on the running time more in the next subsection where we compare SiS with an existing method on very large real data sets.

### Maximal frequent subgraph in global metabolic network

In this section, we employ SiS to find maximal frequent subgraph and compare its performance with MULE [14]. Given, a frequency threshold and a collection of graphs, a maximal frequent subgraph finding algorithm finds the maximal subgraphs over the given threshold. MULE is one such tool. we obtained its executable from the authors. SiS finds the frequent subgraphs of a given size but it is easy to extend it to find maximal frequent subgraphs using the simple technique described at the end of Section 3.2. In this experiment, we used the global network data set and measured the *running time *of SiS and MULE

A maximal frequent subgraph finding algorithm is practical only if it scales with lower frequency threshold and large size of the data set. We conducted experiments with different frequency threshold values and different size of the input data sets (e.g., Eukaryote-G data set (145 organisms) and Prokaryote-G data set (1,486 organisms)).

**Results on the Eukaryote-G data set **In Eukaryote-G data set, we ran MULE and SiS with different frequency threshold values and measured the running time. First, we set the frequency threshold to 83%. MULE ran for 20 minutes to find 925 frequent subgraphs and the largest one had 25 edges. We then reduced the threshold to 80%. MULE ran for seven hours and 27 minutes and found three maximal frequent subgraphs of 31 edges. Note that the running time of MULE increases dramatically with a minor three percent drop in the frequency threshold value. This observation continues to hold as we further reduced the threshold to 70%. MULE failed to complete execution in two days. The increase of running time with lowered frequency threshold value is expected as the method must evaluate more subgraphs as they satisfy the frequency requirement. The above set of experiments demonstrate that MULE does not scale well with lower frequency threshold values in Eukaryote-G data set.

**Figure 10:**
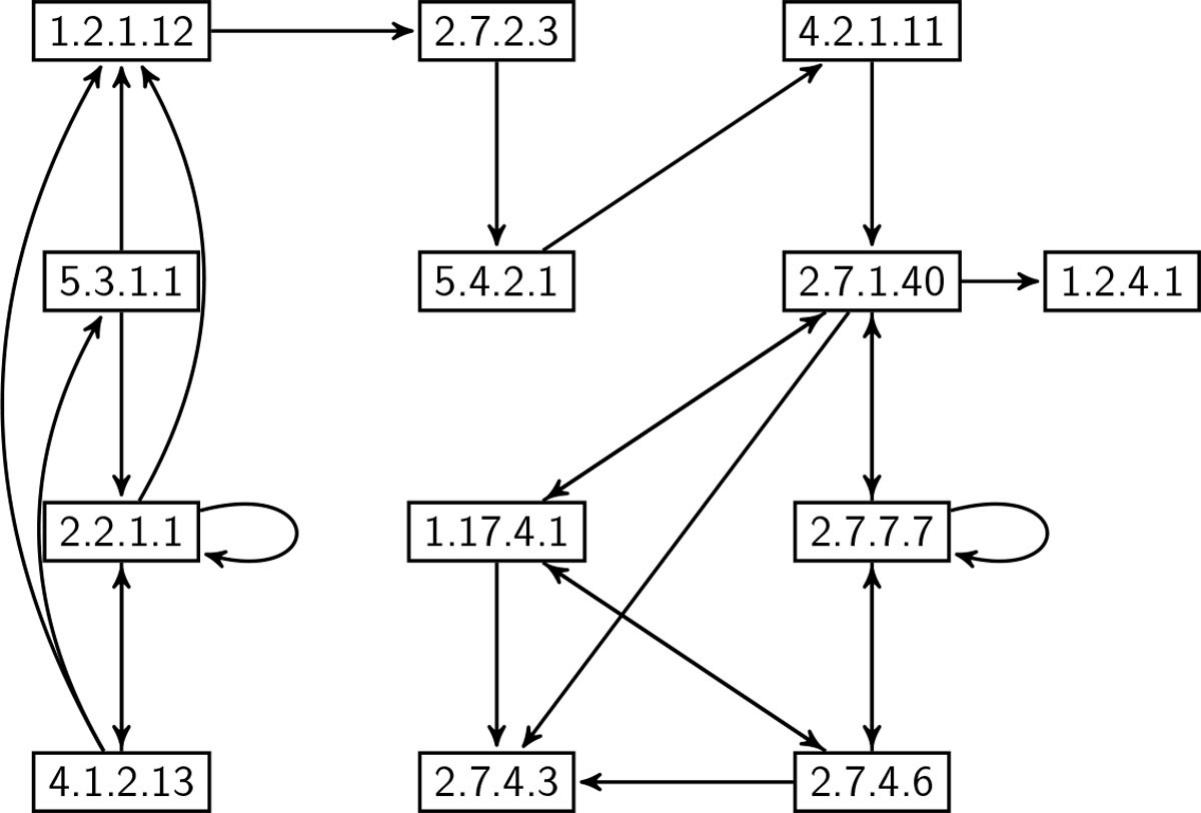
**Maximal frequent size-25 subgraph in Eukaryote-G data set**. Both SiS and MULE identified this subgraph [15].

For frequency threshold of 83% and 80%, we employed SiS to find 50 most probable size-15 and size-20 subgraphs, respectively. We used these subgraphs as seeds and extended them in the greedy manner described before. SiS ran for only 4.25 seconds to generate the same size-25 subgraph found by MULE (Figure 10). SiS ran for four minutes and 18 seconds to generate the same size-31 subgraph found by MULE when threshold was set to 80%. This experiment shows that SiS scales well with the decreasing values of the frequency threshold.

**Results on the Prokaryote-G data set **Next, we compare the performance of SiS and MULE with a larger data set (Prokaryote-G). MULE failed to run on this data set, possibly due to higher memory requirements. SiS ran for 5.38 seconds to generate the maximal frequent subgraph from 50 most probable size-15 subgraphs. It took less than five minutes for SiS to generate the maximal frequent subgraph from most probable size-20 subgraphs. This experiment shows that SiS scales well with the increasing size of the data set.

*These results demonstrate that SiS finds the maximal frequent subgraph orders of magnitude faster than MULE and scales to large data sets where MULE fails*.

### Maximal frequent subgraph in human transcription factor regulatory networks

**Figure 11:**
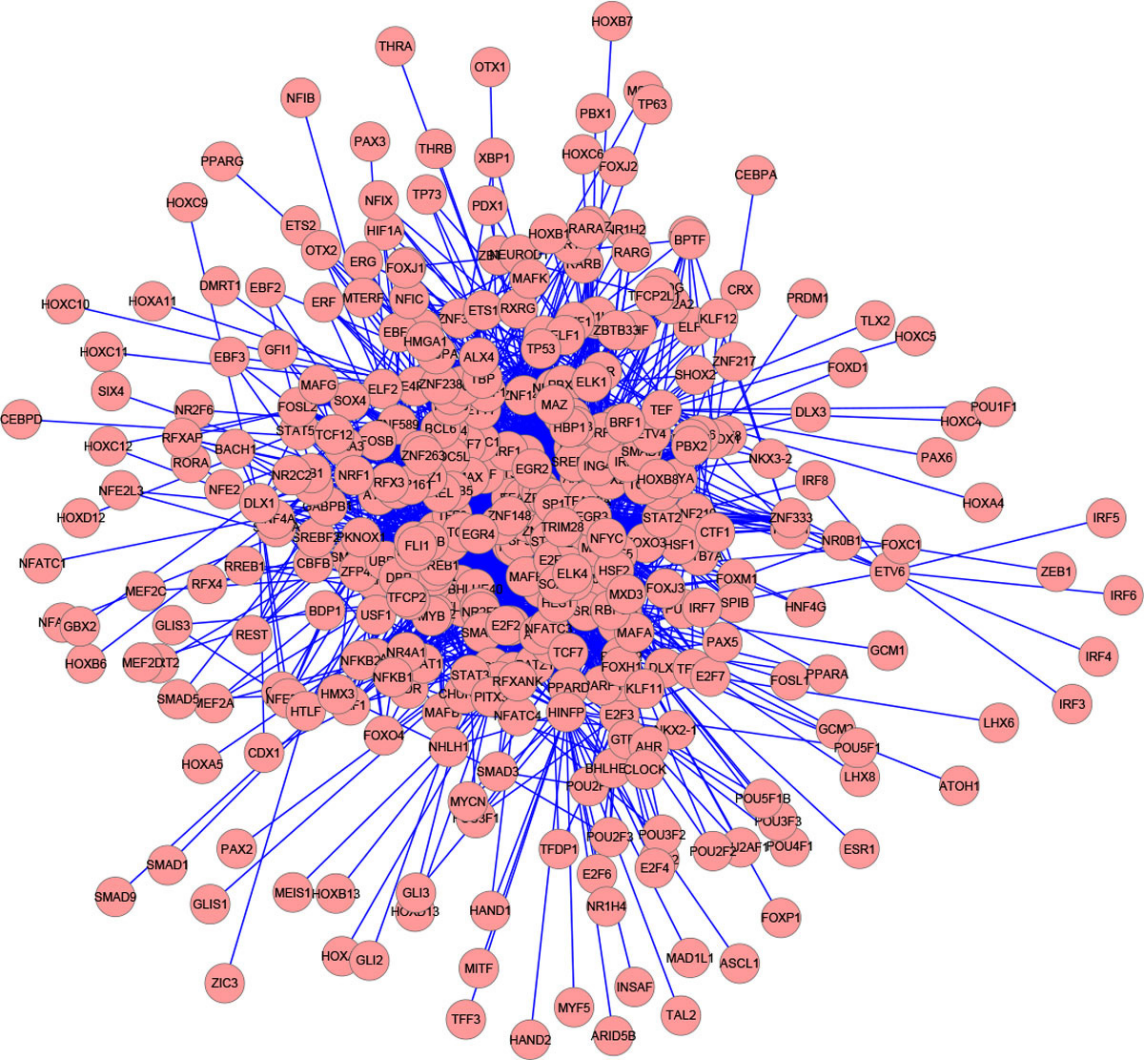
**Maximal frequent subgraph in human transcription regulatory networks found by SiS**. This figure is visualized by Cytoscape.

Interactions between transcription factors comprise a complex regulatory network that defines cellular identity and functions [23]. Sequence-specific transcription factors play a vital role in gene control in eukaryote. For this experiment, we used the human transcription regulatory network data set. On average, each of these networks contains 521 genes and 14,003 interactions. These networks are highly dense. In total, there are 538 unique genes that are interacting with 47,945 unique interactions. This statistics show us that there are some interactions that are unique to specific cell/ tissue types. In addition to this, these networks also have a core regulatory backbone network that are common to all these different networks irrespective of cell/ tissue types. We employed SiS to find maximal frequent subgraph in this data set that is present in at least 70% of these networks. SiS found the maximal frequent subgraph consists of 371 genes and 2382 interactions that satisfies the minimum support over the data set. This massive subgraph hints at the stunning fact that despite diverse cell types, there is a core backbone network among these transcription regulatory networks. Figure 11 shows the maximal frequent subgraph found in this experiment. See Additional file 1 for the interactions in this subgraph.

## Conclusion

We present a method named SiS (Significant Subnetworks) that finds the most probable subgraphs in a large biological network data set. SiS initializes a weighted graph named the template graph that summarizes the input graphs. SiS takes advantage of the template graph while finding the most probable subgraphs of a user-given size, *k*. In other words, SiS finds the subgraphs of *k *interactions with the largest probability to appear in a network selected randomly from the input data set (i.e., size-*k *subgraphs with smallest total edge weights in the template graph). Our experiments comprehensively demonstrate that most probable subgraphs often have very large frequency values as well. Using SiS on metabolic network data set, we found that subgraphs in eukaryote are more frequent than those of prokaryote. We also observed an interesting relationship between the frequency of the subgraphs found by SiS in eukaryote and prokaryote. We performed tests on very large data sets where SiS showed excellent superiority over existing method, MULE. For instance, depending on the size of the subgraph, SiS finishes in a few minutes, whereas running time of MULE ranges from several hours to days on the same data set.

## Competing interests

The authors declare that they have no competing interests.

## Authors' contributions

MMH participated in algorithm development, implementation, data set collection, experiments & analysis of the result and writing of the paper. YK participated in algorithm development, implementation, data set collection, experiments & analysis of the result and writing of the paper. TK participated in algorithm development, experiments & analysis of the result and writing of the paper.

## Supplementary Material

Additional file 1 **Maximal frequent subgraph in human transcription regulatory networks**. This file lists the interactions found in the maximal frequent subgraph in human transcription regulatory network data set. Click here for file

## Declarations

The publication costs for this article were funded by NSF under grant IIS-0845439.

This article has been published as part of *BMC Systems Biology *Volume 7 Supplement 4, 2013: Selected articles from the IEEE International Conference on Bioinformatics and Biomedicine 2012: Systems Biology. The full contents of the supplement are available online at http://www.biomedcentral.com/bmcsystbiol/supplements/7/S4.
